# Multiplexed Microsphere-Based Flow Cytometric Assay to Assess Strain Transcending Antibodies to *Plasmodium vivax* Duffy Binding Protein II Reveals an Efficient Tool to Identify Binding-Inhibitory Antibody Responders

**DOI:** 10.3389/fimmu.2021.704653

**Published:** 2021-10-05

**Authors:** Jéssica R. S. Alves, Fernanda F. de Araújo, Camilla V. Pires, Andréa Teixeira-Carvalho, Barbara A. S. Lima, Letícia M. Torres, Francis B. Ntumngia, John H. Adams, Flora S. Kano, Luzia H. Carvalho

**Affiliations:** ^1^ Molecular Biology and Malaria Immunology, René Rachou Institute, Oswaldo Cruz Foundation (FIOCRUZ), Belo Horizonte, Brazil; ^2^ Integrated Research Group in Biomarkers, René Rachou Institute, Oswaldo Cruz Foundation (FIOCRUZ), Belo Horizonte, Brazil; ^3^ Center for Global Health and Infectious Diseases Research, Department of Global Health, College of Public Health, University of South Florida, Tampa, FL, United States

**Keywords:** multiplex assay, inhibitory antibodies, Duffy binding protein, *Plasmodium vivax*, malaria

## Abstract

Malaria remains a major public health problem worldwide, and *Plasmodium vivax* is the most widely distributed malaria parasite. Naturally acquired binding inhibitory antibodies (BIAbs) to region II of the Duffy binding protein (DBPII), a *P. vivax* ligand that is critical for reticulocyte invasion, are associated with a reduced risk of clinical malaria. Owing to methodological issues in evaluating antibodies that inhibit the DBPII–DARC interaction, a limited number of studies have investigated DBPII BIAbs in *P. vivax*-exposed populations. Based on the assumption that individuals with a consistent BIAb response are characterized by strain-transcending immune responses, we hypothesized that detecting broadly reactive DBPII antibodies would indicate the presence of BIAb response. By taking advantage of an engineered DBPII immunogen targeting conserved DBPII neutralizing epitopes (DEKnull-2), we standardized a multiplex flow cytometry-based serological assay to detect broadly neutralizing IgG antibodies. For this study, a standard *in vitro* cytoadherence assay with COS-7 cells expressing DBPII was used to test for DBPII BIAb response in long-term *P. vivax-*exposed Amazonian individuals. Taken together, the results demonstrate that this DBPII-based multiplex assay facilitates identifying DBPII BIAb carriers. Of relevance, the ability of the multiplex assay to identify BIAb responders was highly accurate when the positivity for all antigens was considered. In conclusion, the standardized DBPII-based flow cytometric assay confirmed that DBPII-BIAb activity was associated with the breadth rather than the magnitude of anti-DBPII antibodies. Altogether, our results suggest that multiplex detection of broadly DBPII-reactive antibodies facilitates preliminary screening of BIAb responders.

## Introduction

In recent years, we have witnessed remarkably increased levels of global funding to combat malaria, which resulted in a significant reduction in the burden of the disease (1). Despite this, the WHO warned that the current progress in the global response to malaria had plateaued, and that key targets of the WHO’s global malaria strategy would likely be missed (2). In particular, *Plasmodium vivax*, the most widespread human malaria parasite (3), poses challenges to control strategies, as this parasite is typically associated with low levels of parasitemia (4–6) and is able to transmit sexual-stage parasites before clinical manifestation (7, 8). Unlike *P. falciparum*, which may use several red blood cell invasion pathways (9), *P. vivax* infects human reticulocytes through a main pathway that requires interaction between an apical parasite protein, the Duffy binding protein (DBP), and its cognate reticulocyte receptor, the Duffy antigen receptor for chemokines (DARC) (10–12). While the receptor-binding domain of DBP (~350 amino acid residues known as region II, DBPII) is the best-characterized and leading blood-stage vaccine candidate against *vivax* malaria (13), the malaria-exposed populations demonstrated DBPII strain-specific immunity (14–16). However, among the same population, it is possible to find a small portion of a stable strain-transcending DBPII inhibitory response (15, 17–19), which is associated with the presence of BIAbs (20) and reduced risk of *P. vivax* clinical malaria (19, 21, 22). These findings suggest that a broadly reactive DBPII inhibitory response should be pursued in all DBPII-based vaccine strategies. In view of this, we recently demonstrated that a second-generation engineered DBPII immunogen lacking most variant strain-specific epitopes (termed DEKnull-2) retained good immunogenicity and induced a broadly reactive BIAb response (20).

*In vitro* evaluation of DBPII antibodies able to block *P. vivax* reticulocyte invasion has proven challenging, as short-term *P. vivax* blood-stage culture is not available for routine use in most malaria research laboratories (23). Consequently, different binding assay platforms have been used to estimate the effects of antibodies to inhibit the DBPII-DARC interaction (24–26). The COS-7 erythrocyte-binding assay is a reference protocol based on the interaction between DBPII expressed on the surface of transfected mammalian COS-7 cells and DARC-positive erythrocytes (27). As antibodies in the COS-7 assay face challenges in inhibiting highly multivalent cell interactions (i.e., DBPII present on surface of COS-7 cells and DARC expressed by RBC), it has been suggested that small differences in antibody activity might not be detected (25). An alternative platform is based on a lower affinity multimer-dimer interaction, in which recombinant DBPII interacts with a DARC-Fc recombinant protein (24, 25). While an assay based on the recombinant DARC-protein seems to be more amenable to high-throughput analysis, an assay with low valency interaction may overestimate inhibitory activity. Even so, *in vitro*-binding assays have greatly contributed to the study of naturally acquired antibody responses able to block the interaction between DBPII and DARC (17–21, 28).

Considering the limitations of routine evaluations of antibodies targeting DBPII-DARC interaction, the rationale was to develop a more “friendly” assay that indicates the presence of BIAbs. As a consistent BIAb response is characterized by DBPII strain-transcending immunity, we hypothesized that simultaneous detection of antibodies against genetically distinct DBPII variants would increase the chances of identifying individuals with BIAb-specific response. In addition to including highly prevalent DBPII variants circulating in the Amazon region, the advantage of our assay was to include a second generation of engineered DBPII immunogen lacking strain-specific epitopes (termed DEKnull-2), whose reactivity was previously associated with broadly reactive BIAb response (20). Using the multiplex assay, it was possible to demonstrate that the specificities to multiple DBPII variants (“breadth”) rather than the magnitude of anti-DBPII antibodies, correlated with BIAb response.

## Material and Methods

### DBPII-Specific Monoclonal Antibodies and Panel of Samples

#### DBPII Monoclonal Antibodies

Two specific DBPII monoclonal antibodies (mAbs) were used to evaluate the ability of the multiplex assay to assess DBPII target epitopes. The 2D10 inhibitory mAb targets disulfide-bonded epitopes, which are critical for the maintenance of the native conformation required for erythrocyte receptor recognition in DBL domains (29, 30). The noninhibitory 3D10 mAb recognizes both denatured and refolded antigens (29).

#### Pooled Plasma Samples

To standardize the multiplexed microsphere-based flow cytometric assay, three types of pooled plasma samples were prepared: (i) DBPII-positive plasma pools obtained from individuals naturally exposed to *P. vivax* who had high levels of anti-DBPII antibody responses as characterized by the presence of BIAb activity (>90% of inhibition in the COS-7 cells) and ELISA-detected antibodies (reactive index >5 for different DBPII constructions); each positive pool included four individual samples; (ii) DBPII-negative pools from malaria endemic area, which were selected from individuals (four samples per pool) living in an endemic area (Amazon Basin) but with no detectable DBPII antibody response (negatives for BIAbs and ELISA); and (iii) DBPII-negative pools from individuals living in a nonendemic area of malaria (Belo Horizonte, Minas Gerais, Brazil) and who have never been exposed to malaria transmission (negative pool nonendemic area).

#### *P. vivax* Plasma Samples

To evaluate the DBPII-multiplexed microsphere-based flow cytometric assay, the study included a total of 245 samples from 85 long-term *P. vivax*-exposed Amazonian individuals. The clinical, immunological, and epidemiological characteristics of the study population have been previously described (31). Briefly, the studied individuals were adults with a similar proportion of males to females (0.95:1), who had been living in the Amazon area for an average of 35 years (IQR: 24–50). As these individuals were Amazonian natives, their age basically corresponds to the time of exposure to malaria (median age, 43 years; IQR: 29–54). Consequently, most individuals did not have circulating parasites (as detected by microscopy or PCR-based assays), and *P. vivax* malaria infection was detected only in two out of 85 (2%) of them (asymptomatic infections).

The ethical and methodological aspects of this study were approved by the Ethical Committee of Research on Human Beings from the Research Institute René Rachou (Report No. 007/2006, No. 07/2009, No. 12/2010, No. 26/2013, and CAAE 50522115.7.0000.5091). The current study was conducted according to the laboratory biosafety and biosecurity policy guidelines of the Fundação Oswaldo Cruz (FIOCRUZ), Brazilian Ministry of Health (http://www.fiocruz.br/biosseguranca/Bis/manuais/biosseg_manuais.html).

### Recombinant DBPII Antigens

The recombinant *P. vivax* DBPII used in this study included amino acids 243–573 of the Sal-1 reference strain (32) and from Brazil-1 (Brz-1), a highly prevalent DBPII variant circulating in the Amazon area (33), and DEKnull-2 (20, 34). All proteins were expressed as 39 kDa 6× His fusion proteins in *Escherichia coli*, as previously described (20, 35).

### ELISA-Detected Antibody Response

A conventional enzyme-linked immunosorbent assay (ELISA) for antigen-specific IgG antibody response was carried out as previously described (36, 37) with plasma samples diluted at 1:100 and DBPII recombinant proteins used at a final concentration of 3 μg/ml. For each protein, the results were expressed as the ELISA reactivity index (RI), calculated as the ratio of the mean optical density (OD_492_) of each sample to the mean OD plus three standard deviations of samples from 20 to 30 unexposed volunteers. RI values >1.0 were considered positive.

### Coupling Beads and Flow Cytometry Assay

The protocol described here was based on a multiplexed microsphere-based flow cytometric assay using the BD™ CBA Human Soluble Protein Flex Set System (BD Biosciences, Franklin Lakes, NJ, USA). This assay provides a method of capturing a set of analytes with beads of known size and fluorescence, allowing the coupling of different antigens to beads with different positions to create a multiplex assay. Here, functional beads (BD Biosciences) were coupled to *P. vivax* recombinant proteins according to the manufacturer’s protocol. Briefly, functional beads were incubated with dithiothreitol (1 M) for 1 h at room temperature. The beads were then washed and resuspended in a coupling buffer (BD Biosciences). Next, recombinant DBPII Sal-1, DBPII Brz-1, or DEKnull-2 at 1 mg/ml were activated by incubation with sulfosuccinimidyl 4-(*N*-maleimidomethyl) cyclohexane-1-carboxylate for 1 h. Next, the activated protein was added to the beads and allowed to conjugate for 1 h at at room temperature. *N*-Ethylmaleimide (2 mg/ml) was added, and the mixture was incubated for 15 min. The beads were then washed and resuspended in storage buffer (BD Biosciences). For assessment of antibody responses, 1 µl of coupled beads were washed and incubated with plasma diluted in 100 µl of washing buffer for 2 h. The unbound antibodies were washed off and then incubated with a biotin-goat anti-human IgG antibody (A18815, Invitrogen, Waltham, MA, USA) and streptavidin-PE (554061, BD Biosciences). Simplex titration assay was performed to determine the best plasma sample dilution using twofold serial dilutions (starting at 1:50) was used in the test. Samples were acquired using a FACSVerse cytometer (Becton Dickinson) equipped with three lasers (405, 488, and 640 nm) capable of distinguishing the flex set of beads through the CBA Red and CBA NIR bead channels. The detection reagent was a phycoerythrin (PE)-conjugated antibody (A18815; Thermo Fisher Scientific, Waltham, MA, USA), which provides a fluorescent signal in proportion to the amount of bound antibodies (reporter channel PE). Antibody response was determined by obtaining the median fluorescence intensity (MFI) values using the FlowJo program (version 10.4.1.), following the analysis strategy described in **Figure S1**. Briefly, the microspheres were selected based on their morphometric features (forward scattering (FSC) and side scattering (SSC)). Next, the CBA-NIR and CBA-Red channels were selected to discriminate each bead population based on the fluorometric profiles. The MFI was determined in each population by selecting histograms of Comp-PE, wherein the *x*-axis represents the intensity of secondary staining. The exact value of MFI was obtained by selecting the statistical features of MFI and Comp-PE.

### DBPII-Transfected COS-7 Cells and Erythrocyte-Binding Assay

The functional properties of DBPII antibodies (BIAbs) were assessed using COS-7 cell erythrocyte binding assays, as previously described (17, 26, 28). Briefly, COS-7 cells (green monkey kidney epithelium; ATCC, Manassas, VA, USA) were transfected with the plasmid pEGFP-DBPII Brz-1 (common DBPII allele/variant circulating in the Amazon area) (33) using Lipofectamine and PLUS reagent (Invitrogen Life Technologies, Carlsbad, CA, USA) according to the manufacturer’s protocol. Briefly, COS-7 cells were seeded in a six-well culture plate (1.5 × 10^5^ cells/well) were transfected with the plasmid (0.5 µg/well) liposome complexes (5% Plus-reagent and 3% lipofectamine) in Dulbecco’s modified Eagle’s medium (DMEM, Sigma-Aldrich, St. Louis, MO, USA) without fetal bovine serum (Gibco-BRL Life Technologies, Rockville, MD, USA). After 6 h of exposure to the DNA-liposome complexes (37°C, 5% CO_2_), the transfection medium was replaced with DMEM supplemented with 10% fetal bovine serum. Twenty-four hours after transfection, the culture medium was replaced again, and the efficiency of transfection was assessed using an inverted fluorescence microscope (Nikon TE2000E, Melville, NY, USA). Forty-eight hours post-transfection, erythrocyte-binding assays were performed as previously described (26). Plasma samples at 1:40 dilution in DMEM without fetal bovine serum were added to plates with transfected COS-7 cells transfected, and plates were incubated for 1 h at 37°C in 5% CO_2_. Human erythrocytes O^+^ DARC^+^ (genotype Fy*A/Fy*B) in a 10% suspension were added to each well (200 µl/well), and the plates were incubated for 2 h at room temperature. After incubation, unbound erythrocytes were removed by washing the wells three times with DMEM medium without fetal bovine serum. Binding was quantified by counting rosettes around GFP-positive cells within 10 fields of view (×200). Binding for each plasma dilution was compared with the binding of transfected COS-7 cells incubated with negative control plasma at a 1:40 dilution (100% binding). The percent inhibition was calculated as 100 × (Rc − Rt)/Rc, where Rc is the average number of rosettes in the control wells and Rt is the average number of rosettes in the test wells. Percent inhibition values were considered positive at >50%. Samples were classified as high responders (HR) when the percentage inhibition values were >90%.

### Statistical Analysis

A database was created using Epidata software (version V2.2.2.183, http://www.epidata.dk). Graphics and analyses were performed using GraphPad Prism version 7 (GraphPad Software, La Jolla, CA, USA, www.graphpad.com) and R statistical software (version 3.3.2). Differences in means and medians were tested using either one-way ANOVA, with Tukey’s *post-hoc* test, the Mann-Whitney test, or Kruskal-Wallis test, with Dunn’s *post-hoc* test, as appropriate. The performance parameters were defined using a 2 × 2 contingency table with 95% confidence intervals (95% CI), calculated using the OpenEpi open-source statistical calculator (openepi.com, Version 3) (38). The performance of the multiplex serological assay was expressed by statistical indices using the results of COS-7 cell erythrocyte binding assays as a reference (gold standard for BIAb response): (i) sensitivity = [true positives/(true positives + false negatives)] × 100; (ii) specificity = [true negatives/(true negatives + false positives)] × 100; (iii) accuracy = [true positives + true negatives/(all sample tests)]. The logistic regression model, followed by odds ratio calculations, with a confidence interval of 95%, was built to describe independent associations between the presence of inhibition by BIAbs and seropositivity against none, one, two, or three recombinant proteins as confounding variables. The dependent variables (BIAb response) of the models were the binary values, where all positive and negative responses were considered “1” and “0,” respectively. To compare the performance between simplex and multiplex versions of multiplexed microsphere-based flow cytometric assays, receiver operating characteristic (ROC) curves were constructed using GraphPad Software. True positive and negative samples were defined according to the presence or absence of BIAb activity, respectively, as determined by the reference methods of the COS7-cell cytoadherence assay (gold standard). The ability of the test to distinguish between positive and negative results was evaluated using the area under the curve (AUC; measure of accuracy).

## Results

### Standardization of DBPII-Multiplexed Microsphere-Based Cytometric Assay

**Figure 1:**
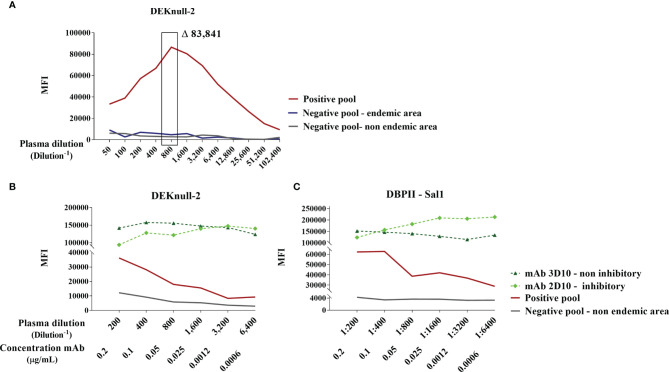
Standardization of DBPII-multiplexed microsphere-based cytometric assay. **(A)** Titration curve for anti-DEKnull-2 antibodies in plasma samples (1:50–102,400) from DBPII-negative pool from individuals living in a nonendemic area of malaria (gray), DBPII-negative pool from an endemic area (blue), and DBPII-positive pool obtained from malaria-exposed individuals who have BIAb response as determined by the reference method of the COS-7 cells (red). The highlighted rectangle represents the selected plasma dilution (1:800). The delta value (Δ) corresponds to the difference in the mean fluorescence intensity (MFI) between the positive pool from malaria-exposed individuals and the pool from nonexposed individuals. **(B, C)** Titration curve of MFI to monoclonal antibodies (mAbs; 0.2–0.006 μg/ml) or plasma-pooled samples (1:200–6,400). Inhibitory mAb 2D10 was represented by a light-green diamond and noninhibitory mAb 3D10 by a dark green triangle. Plasma pooled from individuals living in a nonendemic area are represented with a gray line and positive pooled samples with a red line. The MFI values were obtained using the FlowJo program (version 10.4.1) according to the analysis strategy described in **Figure S1**.

To confirm the coupling of antigens and the ideal conditions to differentiate positive and negative samples in the flow cytometry-based DBPII assay, we first carried out a pilot assay, in which each recombinant protein was evaluated with serial dilutions of well-characterized positive and negative pooled samples. Based on the titration curve of DEKnull-2 (**Figure 1A**), a 1:800 plasma dilution was chosen as the best dilution to discriminate between positive and negative responses. At the same dilution, significant differences between positive and negative pooled samples were also obtained for DBPII Sal-1 (10–11 times) and DBPII Brz-1 (three to four times) (**Figure S2**), which allowed the multiplex assays to be performed using a single plasma dilution.

As recombinant DBPII proteins were covalently bound to beads, we further assessed the availability of surface-exposed epitopes using two DBPII-specific mAbs, including one (mAb 2D10) that recognizes DBPII conformational inhibitory epitopes. Titration of both mAbs (2D10 and 3D10) demonstrated that the process of covalent coupling of the DEKnull-2 (**Figure 1B**) or native DBPII variants (e.g., Sal-1; **Figure 1C**) to the beads did not interfere with the recognition of DBPII-exposed epitopes, including those recognized by the 2D10 mAb.

### Evaluation of *P. vivax* Plasma Samples in the DBPII-Based Multiplex Flow Cytometry Assay

**Figure 2:**
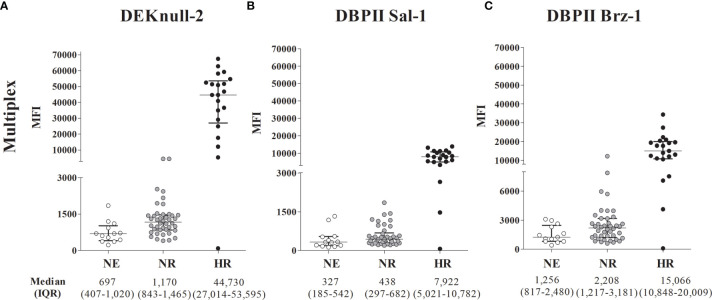
DBPII multiplexed microsphere-based cytometric assay in *Plasmodium vivax* plasma samples from individuals previously characterized as DBPII high responders (HR), non-responders from the malaria-endemic area (NR) or non-exposed (NE). Scatter plots of mean fluorescence intensity (MFI) for individual samples (n = 76) from the endemic area performed with the cytometric bead-based assay multiplex to DEKnull-2 **(A)**, and native DBPII **(B, C)**. The samples were classified according to tercile intervals of reactive index (IR) of ELISA response and inhibitory response (BIAbs) evaluated by COS-7 assay as: High reactive (HR, n = 21), IR ≥ upper tercile and high inhibitory response (black circles); non-responders (NR, n = 42), who presented IR < 1 and negative inhibitory response (dark gray circles) and non-exposed (NE, n = 13), represented by individuals from a non-endemic area (white circles). Median and interquartile range (IQR) values are represented for each group.

For this purpose, plasma samples from the study participants were screened for anti-DBPII antibodies using both conventional ELISA and DBPII-transfected COS-7 cell assay. For the first round of multiplex assays, we selected two subgroups of malaria-exposed individuals (**Table S1**): (i) HR (*n* = 21) who presented with high levels of DBPII antibodies as detected by ELISA (reactivity index ranged 4–22, median 12.10) and BIAb activity (>90% of binding inhibition); (ii) nonresponders from the endemic area (NR, *n* = 42), whose DBPII-specific antibodies (detected by ELISA and BIAbs) were undetectable. In addition, a subgroup of individuals living outside the endemic area and those with undetectable DBPII-specific antibodies were included (nonexposed (NE), *n* = 13). Regardless of the DBPII antigens used in the multiplex assay, the results confirmed that the HR subgroup was clearly discriminated from the NE and NR subgroups (**Figure 2**). For DEKnull-2, the average response of the HR group, as evaluated by mean of fluorescence (MFI), was roughly 38–64 times greater than those of the nonresponder groups (NR and NE, respectively) (Dunn’s test, *p* < 0,05 for all antigens) (**Figure 2**; **Table S2**).

The ROC curves confirmed comparable results between the simplex and multiplex versions of the assay (**Figure S3**), with the ability to distinguish between positive and negative results, with AUC >80% for simplex and >90% for multiplex. More specifically, the minimum differences between the HR subgroup and NE/NR in the simplex version were 18 times for DEKnull-2 and approximately eight times for DBPII Brz-1 or DBPII Sal-1 (**Table S2**).

### Breadth of DBPII IgG Antibody Response in Multiplex Assay and BIAb Response

**Figure 3:**
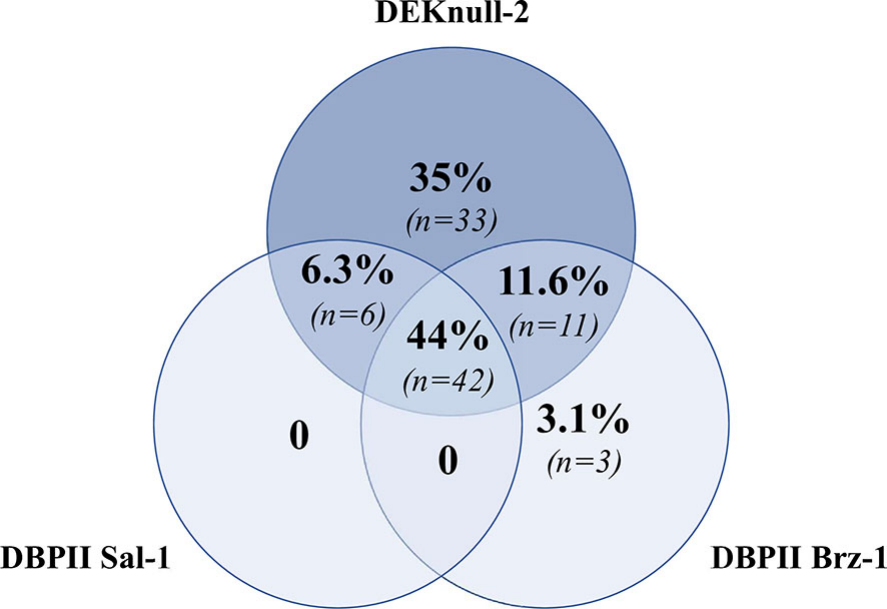
Venn diagram showing overlap positivity for DBPII constructions in the DBPII-based multiplex flow cytometry assay. Venn diagram was built with 95 plasma samples that were positive for at least one DBPII construction in the multiplex assay, which included naturally occurring DBPII variants (DBPII Sal-1 and Brz-1) and the engineered DBPII immunogen (DEKnull-2). Each circle contains the proportion and number of positive samples for DEKnull-2 (dark blue) and either DBPII Sal-1 or DBPII Brz-1 (light blue). Proportions/numbers of samples that were positive for more than one antigen are displayed in circle intersections.

To assess the specificities of detected antibodies for multiple DBPII constructs, a panel of 245 plasma samples from individuals naturally exposed to *P. vivax* was screened for the presence of DBPII-specific antibodies by the multiplex flow cytometry assay. A total of 95 out of 245 (39%) samples were positive for at least one DBPII construct. A Venn diagram showed that a significant number of samples were positive for all assayed antigens (42/95, 44%) (**Figure 3**), confirming the detection of broadly reactive DBPII antibodies. Considering the positivity of each recombinant protein, a high response was detected for DEKnull-2 (34/95, 36%).

**Figure 4:**
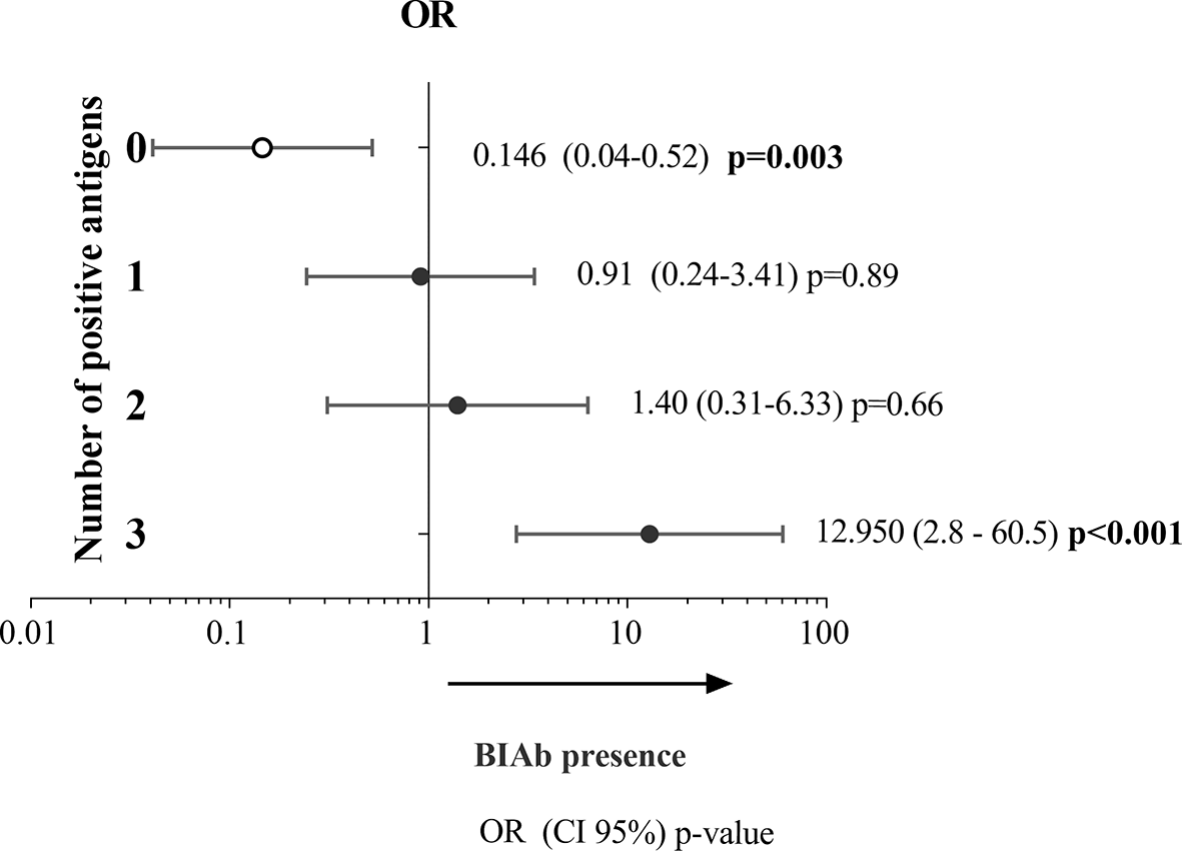
Association between DBPII antibody response as detected in the DBPII-multiplexed microsphere-based cytometric assay and BIAbs as determined by standard COS-7 cytoadherence assay. Two-hundred and forty-five plasma samples of *P. vivax*-exposed individuals were screened for (i) antibodies targeting DBPII-DARC interaction (BIAbs) using the COS-7 cell erythrocyte-binding assays (26) and (ii) anti-DBPII antibody responses as detected by the DBPII-based multiplex assay. The relative likelihoods of positivity in the multiplex assay corresponding to the presence of BIAbs (dependent variable used as reference) were calculated according to the number of DBPII proteins recognized in the DBPII-multiplex assay, including none (zero), one (1), two (2), or three (3). Odds ratio (OR) with respective 95% confidence intervals (95% CI) and *p*-values were determined using the logistic regression model with multiplex seropositivity against none, one, two, or three recombinant proteins used as confounding variables.

To evaluate whether a positive DBPII-multiplex assay would be indicative of the presence of BIAb activity, the abovementioned panel of 245 plasma samples was blindly screened by COS-7 cell erythrocyte-binding assays (**Table S3**). The results demonstrated that 31% (76/245) of the samples presented BIAb activity. Logistic regression models demonstrated that the probability of the DBPII-multiplexed microsphere-based assay to correctly identify the BIAb response increased with the number of antigens recognized in the multiplex assay. Thus, positivity for all three recombinant proteins increased by about 12 times the chances of identifying the BIAb response (OR = 12.95, 95% CI = 2.76–60.59, *p* < 0.001) (**Figure 4**).

As the ability of the DBPII-multiplex assay to identify BIAb responses depended on the number of antigens recognized in the assay, we evaluated the performance of the multiplex assay when identifying BIAb carriers considering the positivity for any antigen (**Figure S4**) as well as for the three antigens assayed in the present study (**Table S1**). The accuracy (AUC) of the multiplex assay was higher when the positivity for all antigens was considered (0.90 *vs*. 0.80), particularly because of an increase in specificity (80% to 97%).

## Discussion

*Plasmodium vivax* DBPII-based vaccines aim to induce broadly neutralizing antibody responses that are able to block merozoite invasion of human reticulocytes (39–41). Although few naturally exposed individuals develop high titers of strain-transcending inhibitory anti-DBPII antibodies (17, 42), these inhibitory antibodies are associated with clinical protection (19, 21). While an *in vitro* DBPII antibody functional assay is an important tool to guide DBPII vaccine development, the detection of DBPII BIAbs in malaria-exposed human populations has been largely restricted (17, 18, 20, 21, 43), perhaps due to the limitation of conducting the current functional assays in the context of larger prospective patient cohorts.

Here, we aimed to develop a conventional multiplex flow cytometry-based serological assay that would indicate the presence of BIAbs as detected by the well-established COS-7 cell transfection-based assay (44). The rationale behind our study was that a simplified methodology to identify potential BIAb carriers would reduce the number of more complex functional assays required to confirm the presence of inhibitory antibodies. The apparent consensus is that multiplex serological assays have advantages in terms of execution time, automation, allowing the use of reduced amounts of biological sample (45). They have been important tools for the simultaneous detection of antibodies against multiple antigens in population-based serological surveys (46–48), especially when vulnerable populations are involved, and a minimal amount of blood should be obtained.

In the simplex version of the DBPII-multiplexed microsphere-based cytometric assay, titration experiments confirmed that this assay correctly differentiated samples showing DBPII-binding activity. In addition, it was possible to suggest that the process of coupling beads does not interfere with the exposure of conformational DBPII epitopes, as both inhibitory (2D10) and noninhibitory (3D10) DBPII mAbs were correctly recognized. This is particularly relevant as the crystal structure of the DBPII/2D10 complex showed that 2D10 targets disulfide bond-dependent DBPII epitopes, which are critical for the maintenance of the native conformation of proteins required for DARC recognition (30). Although a single inhibitory monoclonal antibody was used as a control in our experiments, the 2D10 mAb can bind divergent DBP alleles (30); in particular, the same authors demonstrated that the 2D10 epitopes were invariant in more than 90% of the DBP sequences representing global diversity (49), which confirms that this mAb recognizes broadly conserved epitopes. Furthermore, groups of well-characterized DBPII responders (i.e., HR, NR, and NE) allowed us to assess the antibody response to different DBPII variants in a multiplex format assay. Taken together, using a minimum number of plasma samples, this multiplex detection corroborates previous reports showing the feasibility of multiplex detection of malaria antibody responses against different allelic variants of the same antigen (50, 51).

To investigate whether the DBPII-multiplexed microsphere-based cytometric assay would be indicative of the presence of BIAb activity, we used the COS-7 cell erythrocyte-binding assays to screen a panel of *P. vivax* samples for the presence of antibodies targeting DBPII-DARC interaction. The results demonstrate that the ability of the DBPII-based multiplex assay to correctly identify BIAb activity was associated with the detection of antibodies against both conserved (DEKnull-2) and polymorphic DBPII epitopes (Sal-1 plus Brz-1). Indeed, the odds ratio (OR) statistic showed that positivity for all three recombinant proteins in the multiplex assay increased the chances of identifying BIAb carriers by about 12 times. The accuracy of the multiplex assay to identify BIAb carriers was 90% when multiplex positivity took into account all three antigens. In our multiplex assay protocol, the results demonstrated that the “breadth,” (i.e., the specificities for multiple DBPII variants) rather than the levels of DBPII-based antibodies, was related to the presence of BIAbs. Considering that BIAbs activity is associated with strain-transcending inhibitory immune responses, we can assume that the detection of anti-DBPII antibodies against a range of diverse allelic DBPII variants seems to be more relevant than levels of the antibody *per se* in our multiplex assay. In this scenario, it would be interesting to explore whether the inclusion of additional antigens involved in the reticulocyte invasion would increase the chance of detecting BIAbs; for example, the *P. vivax* reticulocyte-binding protein (PvRBPs) family that are involved in novel reticulocyte invasion pathways (52–54). Also, a distinct erythrocyte-binding protein (EBP2), a new member of the DBP family, which may represent a novel ligand for an alternative pathway of DARC-positive reticulocyte invasion (55).

Remarkably, while Sal-1 and Brz-1 are highly prevalent DBPII variants circulating in the study area (31, 33, 49), we observed a predominance of response to DEKnull-2 over these DBPII-strain-specific antigens. This was not unexpected, as we have previously demonstrated that while antibody responses to DBPII-strain-specific antigens (such as Sal-1 and Brz-1) could be influenced by seasonal variation in malaria transmission (37), the immune response towards the conserved epitopes present in DEKnull-2 was stable and indicative of naturally acquired immunity (56). Consequently, it seems reasonable to speculate that some degree of *P. vivax*-acquired immunity was naturally acquired in the studied individuals with high BIAb activity (>90% as detected here with COS-7 cells). Nevertheless, evaluating the protective nature of the antibody response was outside the scope of the current study. To properly address the clinical protection mediated by BIAbs, a long-term follow-up study is required with an estimated sample size that allows statistical power. Despite this, different studies have already confirmed the association between clinical protection and the presence of BIAb responses (15, 21, 22), including in the Brazilian Amazon area (19).

The current study has limitations that should be taken into account when interpreting the results. First, we did not use purified DBPII IgG antibodies to confirm IgG binding to the beads. However, we used dose-response curves of well-characterized BIAb plasma samples (positive and negative) that confirmed the specificity of the assay using different contrations of highly specific antihuman IgG conjugate. Of relevance, we previously used confocal microscopy to confirm that BIAb-positive plasma samples are characterized by high levels of IgG antibodies targeting DBPII-DARC interaction (28). Furthermore, we titrated two IgG DBPII-specific mAbs. Consequently, we are confident about the specificity of the assay for detecting DBPII IgG antibodies. However, to adapt this protocol to a semiautomated multiplex high-throughput assay, additional assay parameters should be evaluated, including the stability of antigen coupling after different storage conditions, and controls that suffer less from interassay variations such as purified/mono- or polyclonal antibodies. Second, the DBP functional assay used here only demonstrates the inhibition of DBP-DARC binding, but not the most desirable *in vitro* assay available, which is the invasion of human reticulocytes by *ex vivo P. vivax* isolates. We previously used a short-term *ex vivo P. vivax* cultures to demonstrate that anti-DEKnull-2 antibodies were able to inhibit merozoite invasion of human reticulocytes (20). Nevertheless, we agree with others that the interpretation of DBPII-DARC-binding assays should be approached with caution. In 2019, Rawlinson et al. (57) demonstrated that some human DBPII mAbs able to block parasite invasion were ineffective at blocking the DBPII-DARC interaction in a protein-protein assay (binding-inhibition ELISA) (57). While these authors used a protein-protein assay that only uses fragments of DARC, we used the established COS-7 cytoadherence assay based on cell-cell multivalent interaction. At this point, we can speculate that a cell-cell interaction assay may be more realistic for evaluating inhibitory antibody response in a protein-protein assay. Although all *in vitro* functional assays present limitations, DBPII-DARC interaction-based assays were decisive in confirming *P. vivax* clinical immunity mediated by anti-DBP antibodies (15, 19, 21, 22).

In conclusion, the successful protocol described here indicates that a multiplex detection of broadly DBPII-reactive antibodies is a potential approach that could be useful to improve the detection of BIAb carriers in large-population cohort studies. For that, it is essential to adapt the multiplex platform to include allelic DBPII variants prevalent in the study area.

## Data Availability Statement

The original contributions presented in the study are included in the article/**Supplementary Material**. Further inquiries can be directed to the corresponding authors.

## Ethics Statement

The studies involving human participants were reviewed and approved by The Ethical Committee of Research on Human Beings from the Rene Rachou Institute – Fiocruz Minas (Reports No. 007/2006, No. 07/2009, No.12/2010, No. 26/2013, and CAAE 50522115.7.0000.5091), according to the Brazilian National Council of Health. The study participants were informed about the aims and procedures and agreed with voluntary participation through written informed consent. The patients/participants provided their written informed consent to participate in this study.

## Author Contributions

Conception and study design: JA, AT-C, FK, and LC. Fieldwork and recruitment of participants: JA, BL, LT, CP, and FK. Performance of experiments and data analysis: JA, FA, and CP. The drafting of the manuscript: LC, FK, CP, and JA. Provide critical advice and reagents: FN and JA. All authors contributed to the article and approved the submitted version.

## Funding

This work was supported by the National Research Council for Scientific and Technological Development-CNPq (422257/2016-8 by LC), the Research Foundation of Minas Gerais-FAPEMIG (APQ-02625-15 by LC), Programa Fiocruz de Fomento à Inovação: Inova Fiocruz (VPPCB-007-FIO-18-2-33 by FK), and the NIH Research Project Grant Program (R01AI064478 by JA and LC). LC and AT-C are research fellows from CNPq and the Coordination for the Improvement of Higher Education Personnel (CAPES, Finance Code 001). Scholarships were sponsored by CAPES (BASL, JRSA) and CNPq (LC, FA, CP, and LT). The funders had no role in the study design, data collection and analysis, decision to publish, or preparation of the manuscript.

## Conflict of Interest

The authors declare that the research was conducted in the absence of any commercial or financial relationships that could be construed as a potential conflict of interest.

## Publisher’s Note

All claims expressed in this article are solely those of the authors and do not necessarily represent those of their affiliated organizations, or those of the publisher, the editors and the reviewers. Any product that may be evaluated in this article, or claim that may be made by its manufacturer, is not guaranteed or endorsed by the publisher.
