# Supplementary material for: Multiplexed Microsphere-Based Flow Cytometric Assay to Assess Strain Transcending Antibodies to Plasmodium vivax Duffy Binding Protein II Reveals an Efficient Tool to Identify Binding-Inhibitory Antibody Responders
Source: Front Immunol. 2021 Oct 5;12:704653. doi: 10.3389/fimmu.2021.704653 (PMC8523986; doi:10.3389/fimmu.2021.704653)
Supplement: Supplementary file 1 [file DataSheet_1.pdf]

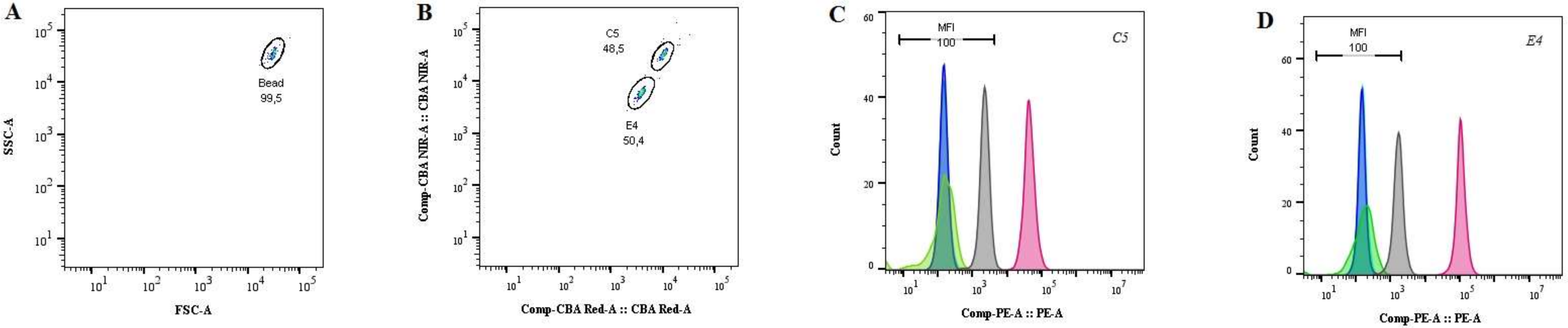

Figure S1: Analysis strategy to determine the mean fluorescence intensity (MFI) on FlowJo program and controls. (A) Analysis strategy on FlowJo to discriminate microsphere populations following the morphometric features selecting (forward scattering [FSC] and side scattering [SSC]), (B) channels CBA-NIR and CBA-Red were selected to discriminate each bead population based on the fluorometric profiles. (C) The MFI was determined in each population by selecting histogram graphs of Comp-PE and the x-axis represents the intensity of secondary staining. The exact value of MFI is obtained by selecting statistical features of MFI and Comp-PE. (C-D) Controls included in each assay are showed; i.e., microsphere-uncoupled (light blue), microsphere-coupled with assay performed in the absence of both plasma sample and anti-human IgG conjugate (dark-blue), microsphere-coupled without plasma but in the presence of conjugate (green). Additional controls included each microsphere population in the presence of negative (gray) and positive (pink) pools.

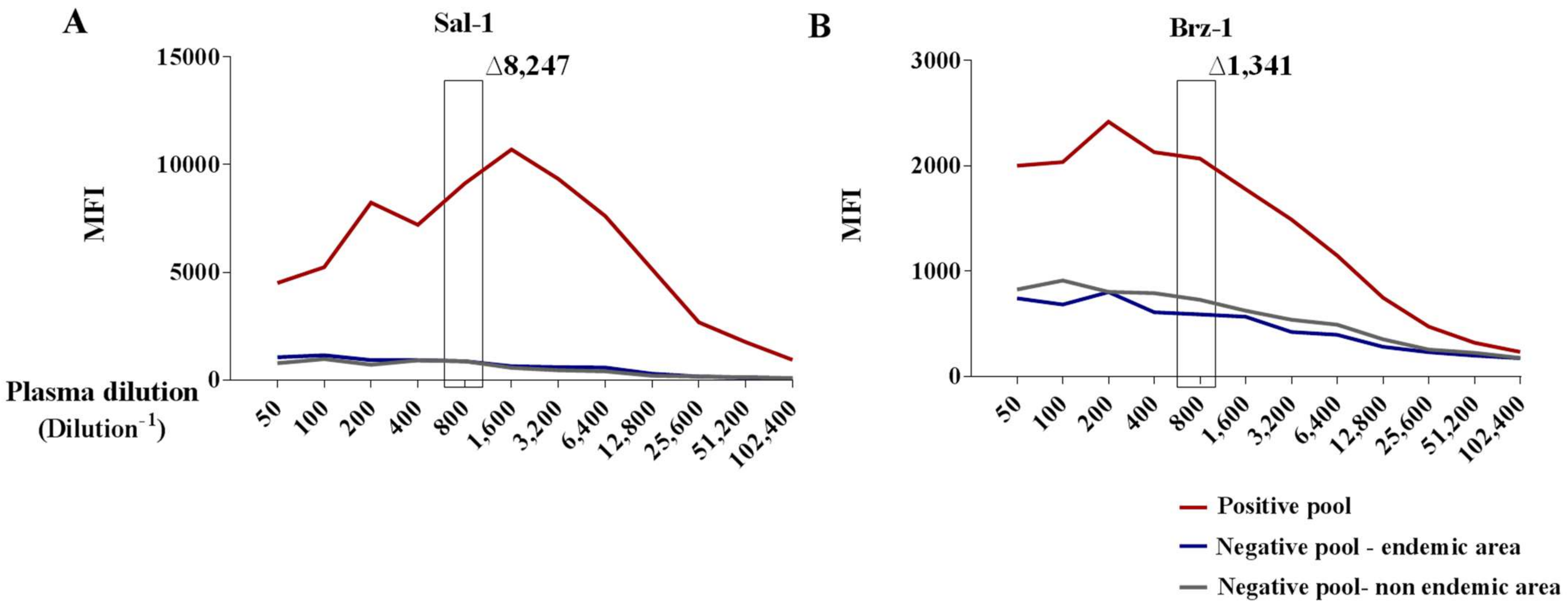

Figure S2: Standardization of DBPII multiplexed microsphere-based cytometric assay. Titration curve for anti-Sal-1 and Brz-1 antibodies in plasma samples (1:50 to 1:102,400). Positive (red) and negative pools (non-endemic area, gray and endemic area, blue) were described in the legend of Figure 1. The highlighted rectangle represents the selected plasma dilution (1:800). The delta value (Δ) corresponds to the difference in the MFI between the positive pool from malaria-exposed individuals and the pool from non-exposed individuals. At 1:800 plasma pooled dilution, MFI values for DBPII Sal-1 were 9,115 (positive pool), 867 (negative pool from endemic area) and 868 (negative pool from non-endemic area); at 1:800 dilution, MIF values for DBPII Brz-1 were 2,069 (positive pool), 590 (negative pool from endemic area) and 728 (negative pool from non-endemic area).

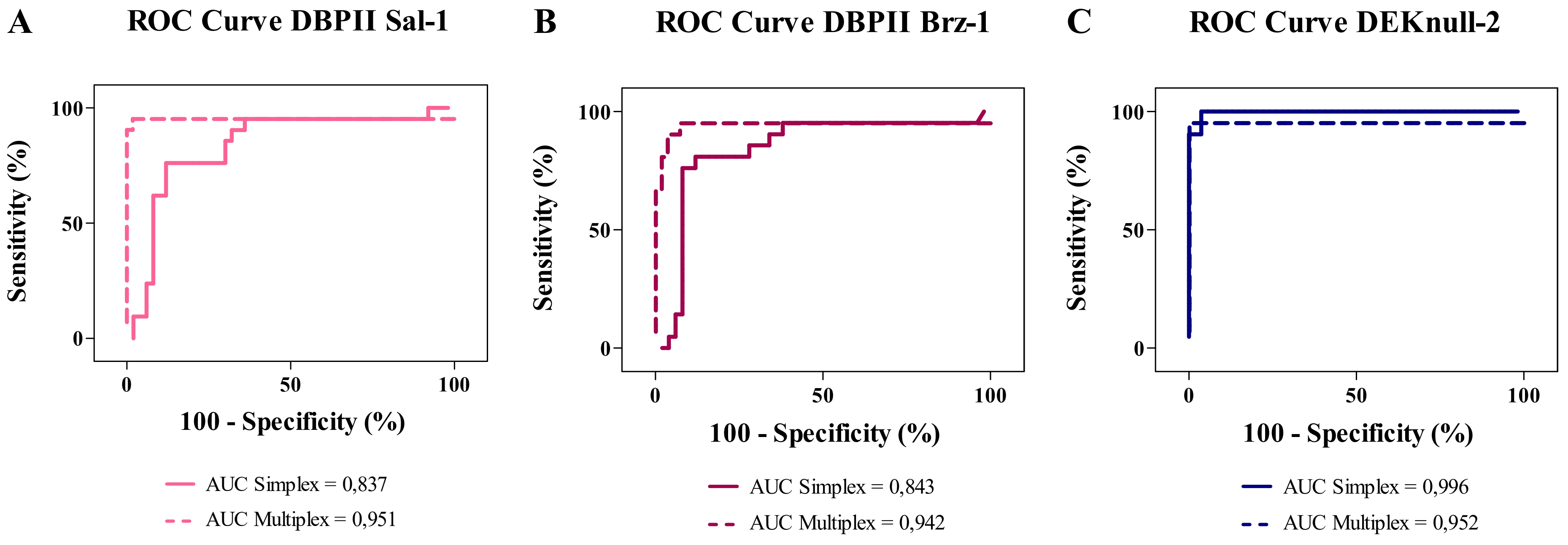

Figure S3: DBPII multiplexed microsphere-based cytometric assay simplex and multiplex performance. Receiver operating characteristic (ROC) plots to compare the performance between simplex and multiplex versions of the DBPII multiplexed microsphere-based cytometric assay to determine the antibody response to DBPII antigens (Sal-1, Brz-1 and DEKnull-2). The analysis was performed including panels of both positive (n=21) and negative samples (endemic area, n=42; and non-endemic area, n=13), as previously described in methods and supplementary Table S2.

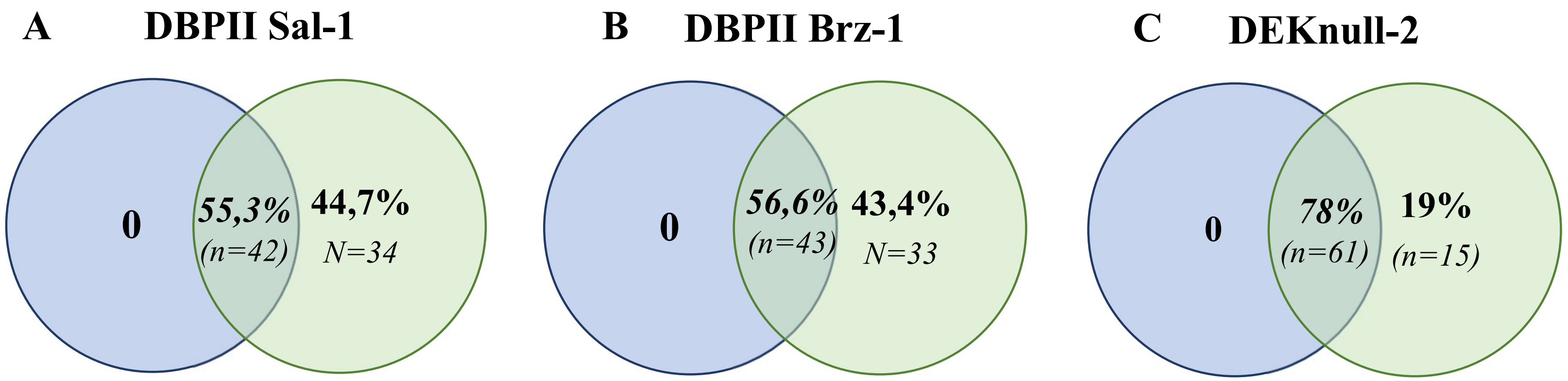

Figure S4: Venn diagram for BIAb positive samples correctly identified by each recombinant protein (Sal-1, Brz-1 and DEKnull-2) in the DBPII multiplexed microsphere-based cytometric assay. Each Venn diagram included all BIAb positive samples (n=76), as detected by the COS-7 assay (green circle). For each protein (green circle), the proportion (number) of BIAb samples identified in the multiplex assay was represented in circle intersections.
