# Supplementary material for: Multiplexed Microsphere-Based Flow Cytometric Assay to Assess Strain Transcending Antibodies to Plasmodium vivax Duffy Binding Protein II Reveals an Efficient Tool to Identify Binding-Inhibitory Antibody Responders
Source: Front Immunol. 2021 Oct 5;12:704653. doi: 10.3389/fimmu.2021.704653 (PMC8523986; doi:10.3389/fimmu.2021.704653)
Supplement: Supplementary file 2 [file DataSheet_2.docx]

**Table S1.**  DBPII ELISA-detected antibody response and inhibitory binding antibody activity (BIAbs) from malaria-exposed individuals grouped as high responders (HR) or non-responder (NR)

| **Group** | **Sample code** | **ELISA RI** | **BIABs (%inhibition)** |
| --- | --- | --- | --- |
| **HIGH RESPONDERS (HR)** | **RP0004** | 5.20 | 99.06 |
|  | **RP0007** | 9.78 | 99.57 |
|  | **RP0021** | 14.80 | 99.34 |
|  | **RP0316** | 13.3 | 99.3 |
|  | **RP0328** | 14.5 | 99.06 |
|  | **RP215** | 7.34 | 98.87 |
|  | **RP251** | 11.8 | 99.17 |
|  | **RP544B** | 10.9 | 99.81 |
|  | **RP571B** | 10.0 | 99.39 |
|  | **RP511B** | 10.8 | 99.67 |
|  | **RP597B** | 10.10 | 99.7 |
|  | **RP534B** | 12.1 | 99.34 |
|  | **RP516B** | 12.1 | 99.62 |
|  | **RP233B** | 4.1 | 93.4 |
|  | **RP544C** | 18.4 | 99.62 |
|  | **RP571C** | 13.4 | 99.7 |
|  | **RP511C** | 18.7 | 99.0 |
|  | **RP597C** | 18.8 | 99.7 |
|  | **RP534C** | 22.0 | 99.38 |
|  | **RP516C** | 20.10 | 99.81 |
|  | **RP233C** | 6.44 | 91.69 |
|  |  |  |  |
| **NON RESPONDERS (NR)** | **RP0210** | 0.15 | 0.00 |
|  | **RP0381** | 0.26 | 0.00 |
|  | **RP0382** | 0.35 | 12.03 |
|  | **RP0402** | 0.98 | 9.40 |
|  | **RP0410** | 0.06 | 14.10 |
|  | **RP0421** | 0.33 | 0.00 |
|  | **RP0437** | 0.19 | 0.00 |
|  | **RP0471** | 0.02 | 0.00 |
|  | **RP0482** | 0.10 | 0.00 |
|  | **RP0490** | 0.00 | 38.7 |
|  | **RP0494** | 0.08 | 0.00 |
|  | **RP0530** | 0.20 | 0.00 |
|  | **RP0550** | 0.34 | 0.00 |
|  | **RP0561** | 0.47 | 0.00 |
|  | **RP210B** | 0.4 | 0.00 |
|  | **RP386B** | 0.24 | 0.00 |
|  | **RP368B** | 0.55 | 17.53 |
|  | **RP102B** | 0.80 | 1.04 |
|  | **RP182B** | 0.13 | 7.60 |
|  | **RP80B** | 0.23 | 16.84 |
|  | **RP154B** | 0.10 | 0.00 |
|  | **RP125B** | 0.05 | 0.00 |
|  | **RP101B** | 0.96 | 3.40 |
|  | **RP114B** | 0.13 | 5.70 |
|  | **RP98B** | 0.32 | 0.00 |
|  | **RP580B** | 0.18 | 0.00 |
|  | **RP413B** | 0.22 | 0.00 |
|  | **RP398B** | 0.25 | 7.88 |
|  | **RP210C** | 0.34 | 0.00 |
|  | **RP386C** | 0.42 | 29.21 |
|  | **RP368C** | 0.74 | 5.87 |
|  | **RP102C** | 0.67 | 0.00 |
|  | **RP182C** | 0.57 | 24.60 |
|  | **RP80C** | 0.37 | 26.81 |
|  | **RP154C** | 0.44 | 0.00 |
|  | **RP125C** | 0.17 | 0.00 |
|  | **RP101C** | 0.08 | 6.40 |
|  | **RP114C** | 0.09 | 0.00 |
|  | **RP98C** | 0.006 | 0.00 |
|  | **RP580C** | 0.27 | 0.00 |
|  | **RP413C** | 4.61 | 0.00 |
|  | **RP398C** | 0.18 | 10.46 |
|  |  |  |  |

ELISA-detected IgG antibody responses were expressed as Reactivity Index (RI), with RI > 1.0 considered positive. Binding inhibitory antibody response (BIAbs) was assessed by DBPII COS-7 assay, with >50% of antibody-mediated inhibition considered positive. High responders (HR, n=21) presented RI (ELISA) ranged from 4 to 22 (median 12.10 RI) and BIABs activity > 90% of inhibition. Non-responders from endemic area (NR, n=42) presented undetectable antibody responses (RI<1 and BIAbs <50%).

**TABLE S2.** DBPII-based multiplex flow cytometry assay carried on simplex and multiplex format

| **Antigen** | **Group** | **SIMPLEX MFI Median (IQR)** | **Ratio SIMPLEX** | **MULTIPLEX MFI Median (IQR)** | **Ratio MULTIPLEX** | **Correlation SIMPLEX x MULTIPLEX r (p value)** |
| --- | --- | --- | --- | --- | --- | --- |
| **DEKnull-2** | **NE** | 1,520 (712-3,727) | HR/NE= **40** | 697 (407-1,020) | HR/NE= **64** | 0,74 (p <0.001) |
|  | **NR** | 3,341 (1,756-6,035) | HR/NR=**18** | 1,170 (843-1,465) | HR/NR=**38** |  |
|  | **HR** | 60,879 (48,586-81,795) |  | 44,730 (27,014-53,595) |  |  |
|  |  |  |  |  |  |  |
| **DBPII Sal-1** | **NE** | 467 (275 - 1,545) | HR/NE= **8** | 327 (185-542) | HR/NE= **24** | 0,46 (p<0.001) |
|  | **NR** | 468 (347-1,103) | HR/NR=**8** | 438 (296-682) | HR/NR=**18** |  |
|  | **HR** | 3,698 (1,261-6,055) |  | 7,922 (5,021-10,782) |  |  |
|  |  |  |  |  |  |  |
| **DBPII Brz-1** | **NE** | 280 (204-568) | HR/NE= **9.7** | 1,256 (816-2,480) | HR/NE=**12** | 0,34 (p=0.0034) |
|  | **NR** | 308 (251-542) | HR/NR=**8.8** | 2,208(1,217-3,181) | HR/NR=**7** |  |
|  | **HR** | 2,719 (1,023-5,658) |  | 15,066 (10,848-20,009) |  |  |

Results of DBPII bead-based cytometry assay (simplex or multiplex format) were expressed in Median Fluorescence Intensity (MFI), and the ratio of high responder (HR) versus non-responders (NR and NE) were estimated to simplex and multiplex (values were represented in bold). The results of simplex and multiplex were correlated by using spearman correlation. Sub-groups (HR, NR and NE) were defined as described in the legend of Table S1.

**TABLE S3.**  Individual data from 245 samples evaluated the DBPII bead-based multiplex flow cytometry assay and DBPII COS-7 assay (BIAbs).

|  |  | **MULTIPLEX ASSAY - MFI** | | |  | **COS-7 Assay** |
| --- | --- | --- | --- | --- | --- | --- |
|  | **Sample code** | **DEKnull-2** | **DBPII Sal-1** | **DBPII Brz-1** |  | **BIAb** |
| **POSITIVE FOR 3 ANTIGENS (N=42)** | RP0004 | **52,215** | **10,215** | **19,365** |  | **99.06** |
|  | RP0007 | **12,102** | **2,654** | **7,086** |  | **99.57** |
|  | RP0018 | **11,380** | **1,701** | **7,917** |  | **99.57** |
|  | RP0021 | **58,166** | **13,135** | **22,306** |  | **99.34** |
|  | RP0137 | **10,005** | **2,004** | **6,303** |  | **98.37** |
|  | RP0142 | **8,586** | **3,686** | **4,846** |  | **95.27** |
|  | RP0145 | **14,763** | **2,429** | **6,348** |  | **99.67** |
|  | RP0215 | **54,674** | **8,616** | **17,729** |  | **98.97** |
|  | RP0223 | **8,486** | **1,791** | **5,045** |  | **99.17** |
|  | RP0260 | **19,978** | **5,036** | **8,962** |  | **99.46** |
|  | RP0267 | **14,449** | **2,984** | **5,724** |  | **100.00** |
|  | RP0316 | **44,706** | **7,793** | **14,082** |  | **99.30** |
|  | RP0328 | **51,457** | **8,859** | **20,968** |  | **99.06** |
|  | RP0388 | **29,751** | **7,038** | **14,060** |  | **99.06** |
|  | RP0569 | **8,393** | **2,745** | **6,116** |  | **99.13** |
|  | RP114C | **4,414** | **1,850** | **12,217** |  | 0.00 |
|  | RP258B | **19,296** | **3,435** | **8,399** |  | **99.10** |
|  | RP258C | **13,583** | **1,950** | **5,449** |  | **98.64** |
|  | RP291B | **20,339** | **2,237** | **6,585** |  | **99.78** |
|  | RP291C | **21,596** | **2,423** | **6,358** |  | **99.56** |
|  | RP308B | **17,203** | **2,822** | **9,078** |  | **99.84** |
|  | RP315B | **6,167** | **2,668** | **5,512** |  | **68.84** |
|  | RP315C | **22,810** | **5,371** | **10,271** |  | **99.56** |
|  | RP511B | **44,730** | **7,922** | **15,066** |  | **99.67** |
|  | RP511C | **36,607** | **5,323** | **10,614** |  | **99.00** |
|  | RP321C | **10,264** | **1,690** | **4,453** |  | **99.00** |
|  | RP514C | **5,771** | **1,920** | **4,285** |  | 0.00 |
|  | RP516B | **50,884** | **10,371** | **17,822** |  | **99.62** |
|  | RP516C | **59,237** | **11,349** | **19,125** |  | **99.81** |
|  | RP527B | **12,380** | **1,996** | **5,045** |  | **85.96** |
|  | RP534B | **62,847** | **11,542** | **27,389** |  | **99.34** |
|  | RP534C | **67,456** | **13,839** | **34,363** |  | **99.38** |
|  | RP544B | **51,718** | **11,193** | **20,401** |  | **99.81** |
|  | RP544C | **46,835** | **8,411** | **19,616** |  | **99.62** |
|  | RP549B | **8,104** | **3,341** | **4,859** |  | **99.38** |
|  | RP549C | **8,846** | **3,566** | **5,904** |  | **99.07** |
|  | RP571B | **24,943** | **5,150** | **12,088** |  | **99.39** |
|  | RP571C | **17,686** | **3,277** | **7,528** |  | **99.70** |
|  | RP597B | **34,898** | **6,023** | **11,081** |  | **99.70** |
|  | RP597C | **40,906** | **6,943** | **13,055** |  | **99.70** |
|  | RP55C | **9,733** | **8,345** | **14,275** |  | 35.49 |
|  | RP607B | **2,538** | **1,652** | **5,134** |  | 0.00 |
| **Positive n (%)** |  | **42 (100)** | **42 (100)** | **42 (100)** |  | **38 (90)** |
|  |  |  |  |  |  |  |
| **POSITIVE FOR 2 ANTIGENS (N=17)** | RP0251 | **29,085** | **4,892** | . |  | **99.17** |
|  | RP0280 | **9,668** | **1,666** | 3,748 |  | **99.39** |
|  | RP246C | **12,517** | **1,935** | 3,878 |  | **100.00** |
|  | RP267B | **9,672** | **1,628** | 3,313 |  | **99.70** |
|  | RP31B | **2,159** | **1,901** | 2,399 |  | 0.00 |
|  | RP32C | **8,412** | **1,650** | 2,842 |  | 34.09 |
|  | RP0294 | **9,804** | 1,431 | **5,314** |  | 41.89 |
|  | RP0319 | **9,917** | 1,556 | **5,085** |  | **99.57** |
|  | RP0424 | **8,108** | 1,246 | **5,049** |  | **99.17** |
|  | RP0437 | **2,527** | 1,392 | **5,705** |  | 0.00 |
|  | RP0536 | **7,483** | 1,383 | **5,224** |  | 4.20 |
|  | RP278B | **8,425** | 1,562 | **5,750** |  | **89.90** |
|  | RP294B | **7,177** | 1,102 | **4,702** |  | 12.52 |
|  | RP386C | **2,166** | 574 | **7,887** |  | 29.21 |
|  | RP428C | **2,904** | 1,582 | **5,111** |  | 0.00 |
|  | RP308C | **7,340** | 1,163 | **4,253** |  | **99.18** |
|  | RP83B | **7,589** | 1,183 | **4,409** |  | **98.80** |
| **Positive n (%)** |  | **17 (100)** | **6 (37.5)** | **11 (64.7)** |  | **9 (53)** |
|  |  |  |  |  |  |  |
| **POSITIVE FOR 1 ANTIGEN (N=36)** | RP0020 | **2,559** | 982 | 3,495 |  | 0.00 |
|  | RP0052 | **9,219** | 1,028 | 3,362 |  | **100.00** |
|  | RP0072 | **5,667** | 1,169 | 2,500 |  | 41.08 |
|  | RP0148 | **9,400** | 1,576 | 4,019 |  | **99.14** |
|  | RP0216 | **4,294** | 934 | 2,099 |  | **99.06** |
|  | RP0254 | **2,220** | 577 | 1,165 |  | 46.98 |
|  | RP0304 | **2,895** | 679 | 2,784 |  | 49.20 |
|  | RP0519 | **3,640** | 523 | 2,518 |  | 15.78 |
|  | RP0545 | **2,745** | 926 | 3,609 |  | 0.00 |
|  | RP101C | **2,437** | 370 | 967 |  | 6.40 |
|  | RP233C | **5,392** | 1,471 | . |  | **91.69** |
|  | RP240C | **2,582** | 1,572 | 3,976 |  | 0.00 |
|  | RP262B | **4,172** | 927 | 2,384 |  | **99.67** |
|  | RP262C | **5,146** | 712 | 1,982 |  | **67.00** |
|  | RP267C | **6,577** | 984 | 2,540 |  | **99.39** |
|  | RP31C | **2,392** | . | 3,025 |  | 4.85 |
|  | RP32B | **3,693** | 1,054 | 2,149 |  | 47.80 |
|  | RP370C | **2,133** | 1,315 | 3,556 |  | 0.80 |
|  | RP413C | **4,454** | 1,434 | 2,435 |  | 0.00 |
|  | RP428B | **2,413** | 902 | 3,782 |  | 0.00 |
|  | RP432C | **2,303** | 541 | 1,585 |  | 0.00 |
|  | RP459B | **3,908** | 516 | 2,638 |  | 39.33 |
|  | RP459C | **3,774** | 501 | 2,368 |  | 35.77 |
|  | RP502B | **5,192** | 1,150 | 3,388 |  | **92.10** |
|  | RP513B | **6,483** | 1,081 | 3,733 |  | **97.25** |
|  | RP513C | **5,892** | 991 | 3,971 |  | **97.71** |
|  | RP514B | **2,319** | 1,580 | 4,004 |  | 0.00 |
|  | RP517B | **3,735** | 822 | 1,822 |  | **87.30** |
|  | RP517C | **2,144** | 469 | 1,249 |  | **80.84** |
|  | RP527C | **7,261** | 1,213 | 2,657 |  | **82.61** |
|  | RP54B | **9,527** | 1,042 | 2,496 |  | **100.00** |
|  | RP54C | **4,647** | 553 | 1,574 |  | **100.00** |
|  | RP61B | **2,180** | 353 | 821 |  | 12.40 |
|  | RP543B | 2,038 | 441 | **4,885** |  | 0.00 |
|  | RP543C | 781 | 226 | **13,888** |  | 0.00 |
|  | RP80B | 1,914 | 529 | **5,965** |  | 16.84 |
| **Positive n (%)** |  | **33 (92)** | **0 (0)** | **3 (8)** |  | **15 (42)** |
|  |  |  |  |  |  |  |
| **NEGATIVE SAMPLES (N=150)** | RP0054 | 1,653 | 272 | 763 |  | **90.64** |
|  | RP0086 | 1,295 | 410 | 926 |  | **68.11** |
|  | RP0108 | 839 | 194 | 625 |  | **57.55** |
|  | RP0111 | 1,002 | 127 | 342 |  | **76.64** |
|  | RP0121 | 1,542 | 810 | 1,528 |  | **51.20** |
|  | RP0289 | 1,218 | 354 | 1,229 |  | **99.39** |
|  | RP0529 | 1,231 | 265 | 1,856 |  | **61.00** |
|  | RP0571 | 1,770 | 262 | 1,343 |  | **86.90** |
|  | RP233B | 89 | 68 | . |  | **93.40** |
|  | RP379C | 1,159 | 395 | 2,029 |  | **51.11** |
|  | RP546B | 1,301 | 652 | 1,267 |  | **63.60** |
|  | RP546C | 1,201 | 563 | 1,298 |  | **61.60** |
|  | RP553B | 921 | 188 | 436 |  | **51.64** |
|  | RP55B | 1,062 | 196 | 548 |  | **86.20** |
|  | RP0005 | 298 | 169 | 436 |  | 0.00 |
|  | RP0016 | 1,921 | 496 | 3,840 |  | 0.00 |
|  | RP0022 | 1,979 | 540 | 3,560 |  | 0.00 |
|  | RP0023 | 1,703 | 915 | 2,777 |  | 0.00 |
|  | RP0029 | 771 | 234 | 1,572 |  | 0.30 |
|  | RP0041 | 200 | 141 | 275 |  | 6.40 |
|  | RP0069 | 306 | 183 | 545 |  | 7.00 |
|  | RP0076 | 790 | 616 | 1,141 |  | 22.42 |
|  | RP0101 | 245 | 90 | 312 |  | 47.26 |
|  | RP0102 | 646 | 349 | 745 |  | 0.16 |
|  | RP0107 | 255 | 62 | 250 |  | 4.25 |
|  | RP0118 | 961 | 179 | 618 |  | 0.00 |
|  | RP0141 | 1,015 | 932 | 1,619 |  | 24.11 |
|  | RP0149 | 1,393 | 581 | 2,521 |  | 30.93 |
|  | RP0210 | 1,458 | 770 | 2,651 |  | 0.00 |
|  | RP0219 | 428 | 288 | 647 |  | 13.00 |
|  | RP0236 | 1,200 | 336 | 1,185 |  | 43.94 |
|  | RP0247 | 523 | 341 | 881 |  | 21.70 |
|  | RP0268 | 1,075 | 678 | 1,519 |  | 12.89 |
|  | RP0290 | 229 | 121 | 389 |  | 0.00 |
|  | RP0297 | 1,604 | 705 | 3,007 |  | 0.00 |
|  | RP0302 | 1,137 | 289 | 1,701 |  | 1.82 |
|  | RP0308 | 535 | 200 | 605 |  | 0.00 |
|  | RP0330 | 1,539 | 280 | 1,907 |  | 0.00 |
|  | RP0338 | 898 | 264 | 1,426 |  | 0.00 |
|  | RP0350 | 675 | 367 | 668 |  | 0.00 |
|  | RP0369 | 1,288 | 296 | 2,332 |  | 0.00 |
|  | RP0381 | 404 | 216 | 723 |  | 0.00 |
|  | RP0382 | 1,309 | 530 | 3,342 |  | 12.03 |
|  | RP0396 | 1,281 | 527 | 1,973 |  | 0.00 |
|  | RP0402 | 1,272 | 322 | 2,680 |  | 9.40 |
|  | RP0410 | 707 | 471 | 1,427 |  | 14.10 |
|  | RP0412 | 590 | 246 | 695 |  | 31.15 |
|  | RP0421 | 465 | 266 | 766 |  | 0.00 |
|  | RP0471 | 958 | 353 | 2,417 |  | 0.00 |
|  | RP0482 | 1,364 | 248 | 841 |  | 0.00 |
|  | RP0490 | 1,303 | 1,139 | 1,918 |  | 38.70 |
|  | RP0494 | 934 | 410 | 3,194 |  | 0.00 |
|  | RP0530 | 1,316 | 404 | 2,356 |  | 0.00 |
|  | RP0543 | 1,469 | 536 | 2,990 |  | 0.00 |
|  | RP0550 | 1,424 | 974 | 2,381 |  | 0.00 |
|  | RP0561 | 653 | 222 | 622 |  | 0.00 |
|  | RP0577 | 1,360 | 328 | 2,354 |  | 34.70 |
|  | RP0707 | 1,082 | 326 | 2,081 |  | 16.58 |
|  | RP101B | 1,108 | 246 | 872 |  | 3.40 |
|  | RP102B | 998 | 238 | 1,077 |  | 1.04 |
|  | RP102C | 981 | 290 | 1,381 |  | 0.00 |
|  | RP114B | 1,437 | 1,169 | 2,341 |  | 5.70 |
|  | RP125B | 566 | 344 | 1,000 |  | 0.00 |
|  | RP125C | 508 | 299 | 844 |  | 0.00 |
|  | RP154B | 1,946 | 1,203 | 3,975 |  | 0.00 |
|  | RP154C | 1,525 | 941 | 3,176 |  | 0.00 |
|  | RP179B | 184 | 93 | 234 |  | 9.42 |
|  | RP179C | 267 | 104 | 395 |  | 6.73 |
|  | RP182B | 848 | 564 | 1,777 |  | 7.60 |
|  | RP182C | 961 | 510 | 1,242 |  | 24.60 |
|  | RP208B | 1,748 | 755 | 3,189 |  | 6.40 |
|  | RP208C | 975 | 231 | 1,731 |  | 0.00 |
|  | RP210B | 1,126 | 653 | 1,836 |  | 0.00 |
|  | RP210C | 882 | 538 | 1,426 |  | 0.00 |
|  | RP215B | 791 | 274 | 746 |  | 18.99 |
|  | RP215C | 711 | 266 | 623 |  | 0.00 |
|  | RP236B | 1,524 | 481 | 979 |  | 6.38 |
|  | RP236C | 1,732 | 761 | 1,770 |  | 0.00 |
|  | RP240B | 1,644 | 1,252 | 2,163 |  | 0.51 |
|  | RP245B | 1,042 | 658 | 1,278 |  | 48.18 |
|  | RP245C | 1,395 | 864 | 1,877 |  | 0.20 |
|  | RP265B | 1,131 | 394 | 1,271 |  | 17.16 |
|  | RP265C | 723 | 326 | 808 |  | 14.07 |
|  | RP277B | 229 | 122 | 436 |  | 0.00 |
|  | RP277C | 198 | 104 | 390 |  | 0.00 |
|  | RP285B | 1,286 | 401 | 2,457 |  | 3.23 |
|  | RP285C | 1,470 | 324 | 2,268 |  | 0.00 |
|  | RP295B | 1,979 | 807 | 3,567 |  | 17.40 |
|  | RP295C | 1,625 | 712 | 2,956 |  | 3.68 |
|  | RP304B | 1,469 | 373 | 2,495 |  | 0.00 |
|  | RP304C | 1,474 | 429 | 2,006 |  | 22.00 |
|  | RP316B | 1,149 | 304 | 1,830 |  | 23.19 |
|  | RP316C | 1,350 | 353 | 1,816 |  | 31.82 |
|  | RP317B | 671 | 524 | 1,057 |  | 4.50 |
|  | RP317C | 670 | 452 | 1,151 |  | 0.00 |
|  | RP321B | 2,039 | 543 | 2,185 |  | 0.00 |
|  | RP340B | 827 | 258 | 1,296 |  | 0.00 |
|  | RP340C | 525 | 195 | 799 |  | 0.00 |
|  | RP348B | 2,052 | 988 | 3,317 |  | 0.00 |
|  | RP348C | 1,949 | 841 | 2,887 |  | 0.00 |
|  | RP365B | 1,290 | 273 | 2,294 |  | 0.00 |
|  | RP365C | 1,125 | 263 | 1,919 |  | 0.00 |
|  | RP368B | 869 | 393 | 1,684 |  | 17.53 |
|  | RP368C | 1,121 | 467 | 2,615 |  | 5.87 |
|  | RP36B | 323 | 189 | 610 |  | 18.00 |
|  | RP36C | 469 | 188 | 971 |  | 12.00 |
|  | RP370B | 1,234 | 381 | 2,278 |  | 0.00 |
|  | RP379B | 1,113 | 314 | 1,883 |  | 2.22 |
|  | RP386B | 746 | 259 | 2,280 |  | 0.00 |
|  | RP398B | 1,021 | 287 | 2,084 |  | 7.88 |
|  | RP398C | 1,494 | 378 | 4,009 |  | 10.46 |
|  | RP405B | 843 | 176 | 813 |  | 43.00 |
|  | RP405C | 788 | 186 | 757 |  | 42.00 |
|  | RP413B | 1,435 | 1,014 | 2,136 |  | 0.00 |
|  | RP442B | 990 | 262 | 1,436 |  | 20.59 |
|  | RP442C | 1,434 | 393 | 2,317 |  | 4.40 |
|  | RP462B | 1,196 | 322 | 2,157 |  | 11.50 |
|  | RP462C | 1,095 | 320 | 2,214 |  | 13.10 |
|  | RP498B | 1,556 | 472 | 2,593 |  | 16.15 |
|  | RP498C | 1,643 | 457 | 2,782 |  | 2.70 |
|  | RP512B | 1,976 | 846 | 2,426 |  | 22.41 |
|  | RP512C | 1,733 | 791 | 2,657 |  | 1.01 |
|  | RP542B | 1,343 | 348 | 1,918 |  | 7.68 |
|  | RP542C | 1,131 | 258 | 1,587 |  | 3.79 |
|  | RP547B | 420 | 207 | 464 |  | 0.00 |
|  | RP547C | 552 | 233 | 793 |  | 0.00 |
|  | RP548B | 472 | 123 | 343 |  | 0.00 |
|  | RP548C | 688 | 150 | 839 |  | 0.00 |
|  | RP550B | 1,408 | 1,230 | 2,498 |  | 2.02 |
|  | RP550C | 992 | 904 | 1,556 |  | 5.43 |
|  | RP553C | 885 | 190 | 476 |  | 12.10 |
|  | RP554B | 276 | 70 | 241 |  | 0.00 |
|  | RP554C | 280 | 72 | 243 |  | 37.82 |
|  | RP569B | 859 | 368 | 678 |  | 6.73 |
|  | RP569C | 730 | 303 | 580 |  | 9.20 |
|  | RP580B | 1,214 | 488 | 2,591 |  | 0.00 |
|  | RP580C | 829 | 346 | 1,387 |  | 0.00 |
|  | RP581B | 366 | 256 | 551 |  | 21.00 |
|  | RP581C | 305 | 127 | 604 |  | 18.70 |
|  | RP615B | 535 | 82 | 254 |  | 0.75 |
|  | RP615C | 489 | 106 | 303 |  | 0.00 |
|  | RP61C | 755 | 218 | 1,016 |  | 0.00 |
|  | RP624B | 1,176 | 434 | 1,548 |  | 15.00 |
|  | RP624C | 867 | 404 | 1,296 |  | 3.87 |
|  | RP759C | 1,354 | 765 | 3,182 |  | 0.00 |
|  | RP80C | 1,486 | 440 | 3,977 |  | 26.81 |
|  | RP88B | 418 | 172 | 793 |  | 0.00 |
|  | RP88C | 341 | 135 | 568 |  | 0.00 |
|  | RP98B | 1,264 | 437 | 3,514 |  | 0.00 |
|  | RP98C | 424 | 201 | 1,143 |  | 0.00 |
| **Positive n (%)** | **0 (0)** | **0 (0)** | **0 (0)** | **0 (0)** |  | **14 (9)** |

Results of DBPII bead-based cytometry assay were expressed as Median Fluorescence Intensity (MFI) calculated on FlowJo program (details of analysis strategy described in Figure S1). Samples were grouped according to the number of positive antigens on multiplex assay (3, 2, or 1). For multiplex assay the cut-off values were determined based on media + 3SD of antibody response of 13 individuals non-exposed, living in non-endemic area. BIAbs response (as determined by DBPII COS-7 assay) was expressed as a percent of inhibition considering a positive response when the percent of inhibition >50%. Positive samples were highlighted in bold. The number (n) and frequency (%) of positive samples to each group are expressed at the bottom.
